# Pretending to Be Better Than They Are? Emotional Manipulation in Imprisoned Fraudsters

**DOI:** 10.3389/fpsyg.2021.562269

**Published:** 2021-03-05

**Authors:** Qianglong Wang, Zhenbiao Liu, Edward M. Bernat, Anthony A. Vivino, Zilu Liang, Shuliang Bai, Chao Liu, Bo Yang, Zhuo Zhang

**Affiliations:** ^1^School of Criminal Justice, China University of Political Science and Law, Beijing, China; ^2^School of Foreign Language, Taishan University, Tai’an, China; ^3^Department of Psychology, University of Maryland, College Park, College Park, MD, United States; ^4^State Key Laboratory of Cognitive Neuroscience and Learning, IDG/McGovern Institute for Brain Research, Beijing Normal University, Beijing, China; ^5^Center for Collaboration and Innovation in Brain and Learning Sciences, Beijing Normal University, Beijing, China; ^6^Beijing Key Laboratory of Brain Imaging and Connectomics, Beijing Normal University, Beijing, China; ^7^Department of Psychology, Nanjing University, Nanjing, China; ^8^School of Sociology, China University of Political Science and Law, Beijing, China

**Keywords:** imprisoned fraudsters, emotional manipulation, psychopathy, MEOS, emotion recognition, empathy

## Abstract

Fraud can cause severe financial losses and affect the physical and mental health of victims. This study aimed to explore the manipulative characteristics of fraudsters and their relationship with other psychological variables. Thirty-four fraudsters were selected from a medium-security prison in China, and thirty-one healthy participants were recruited online. Both groups completed an emotional face-recognition task and self-report measures assaying emotional manipulation, psychopathy, emotion recognition, and empathy. Results showed that imprisoned fraudsters had higher accuracy in identifying fear and surprise faces but lower accuracy in identifying happiness than controls (*t* = 5.26, *p* < 0.001; *t* = 2.38, *p* < 0.05; *t* = 3.75, *p* < 0.001). Significantly lower scores on non-prosocial factors on the Managing the Emotions of Others scale (MEOS) were found for imprisoned fraudsters, relative to controls (*t* = 3.21, *p* < 0.01). Imprisoned fraudsters had low scores in the assessment of psychopathy than the control group, especially Factor 1 (*t* = 2.04, *p* = 0.05). For empathy, imprisoned fraudsters had significantly higher scores in perspective-taking than controls (*t* = 2.03, *p* = 0.05). Correlation analyses revealed that psychopathic traits were positively correlated with non-prosocial factors in both groups. However, the relationships between emotional manipulation and emotional recognition and empathy were not consistent across the groups. The results suggest that fraudsters may pretend to be as prosocial as healthy controls, who had lower antisocial tendencies, normal empathy ability, and would like to manipulate others’ emotions positively during social interaction.

## Introduction

Fraud has become a common type of crime that often has a devastating impact on the victims’ quality of life (Friedrichs, 2009). In Button’s (Button et al., 2014) fraud victims profile, most respondents reported suffering from emotional distress: anger (68.4%) and stress (44.3%). Their financial loss was also significant: 62.7% of victims had a loss of more than £1000. Among fraud cases in China, telecom fraud is predominant, is carried out with minimal contact between the fraudster and the victim, and is mainly prevented by improving Telenet security. However, in our survey of Chinese prisoners, nearly one in three people have a record of face-to-face fraud offenses (contract fraud or fraud between friends or relatives). The consequences of such face-to-face fraud, though less prevalent than telecom fraud, are just as severe and should not be ignored. Moreover, this kind of fraud is more overtly deceptive and can be more difficult to prevent because its perpetrators are often people the victims know and trust. The characteristics that allow face-to-face fraudsters to persuade their victims are unknown, and understanding these characteristics is critical to preventing future crimes.

Previous research has found that fraudsters show great narcissism and psychopathy and exhibit antisocial behaviors (deception, cheating, manipulation, and aggression) (Perri, 2011; Brody et al., 2012). Other research has shown that fraudsters lack social conscientiousness, even if they seem outgoing and agreeable (Collins and Schmidt, 1993; Nee et al., 2019). According to Wolfe and Hermanson’s (2004) Fraud Diamond model, four elements, *incentive*, *opportunity*, *rationalization*, and *capability*, are the essential components of fraudulent behaviors (Wolfe and Hermanson, 2004). In particular, *capability*, which includes factors such as successful lying, having a position of power, intelligence, high confidence, persuasiveness, and affective self-regulation, plays a critical role in a fraudster’s ability to recognize and capitalize on an opportunity for fraud.

Among these capabilities, successful lying is an essential factor for fraud (Barnard, 2008). A fraudster who wants to avoid detection should have an excellent ability to perceive victims’ feelings during face-to-face communication. For example, offender A (one of our participants), who masqueraded as a government official, told the victim that he could help buy a house at a low price. However, after he got the deposit from the victim, he did not keep his promise. Imagine this scam: the victim would not directly tell him, “I believe you” initially. Offender A needed to know the victim’s state in real-time and continuously observed the victim to determine whether he has gained trust. If not, he may need to provide the victim with more evidence that he can be trusted. As we know, facial expression is the primary means of expressing one’s feelings. In other words, the fraudster should recognize the victim’s expression well when committing fraud. Based on the correct understanding of the victim’s emotion, fraudsters can respond according to their purpose and successfully persuade the victims. This emotional sensitivity to others has indeed been found in fraudsters (Möller, 2009; Krokoszinski and Hosser, 2016; Krokoszinski et al., 2018). However, these researches did not specify the emotion type and used only self-reports to measure the capacity to recognize emotions in incarcerated fraudsters, limiting its validity. The research on other incarcerated groups has different emotion recognition results, especially those with psychopathic characteristics (Dawel et al., 2012; Künecke et al., 2018). In the current study, a picture system containing different emotional faces was used to measure fraudsters’ ability to recognize emotions.

Empathy is an essential factor closely associated with the perception of others’ emotions that should also be considered (Eisenberg and Strayer, 1990). The definition of empathy is the ability to understand, experience, and think about others’ feelings (Decety and Jackson, 2004). According to the definition, empathy contains two different components integrating the affective and cognitive aspects (Davis, 1980; Decety and Meyer, 2008). Affective empathy relates to an individual’s ability to share the emotional state of others. Cognitive empathy involves understanding others’ experiences from their facial expressions. Previous studies have demonstrated a close relationship between empathy and facial expression recognition (Besel and Yuille, 2010; Svetieva and Frank, 2016). Deficits of empathy have been found in various offenders (Seidel et al., 2013; Gonzalez-Gadea et al., 2014). However, research on fraudsters’ empathy is still scarce. We can infer that individuals with high empathy will demonstrate emotional perception that would contribute to successful fraud. However, whether empathy would affect recognition of emotion, contribute to fraudulent behaviors, and make their crimes easier, is unknown.

Additionally, successful fraud also requires individuals to manage their own emotions, including suppressing their feelings and controlling emotional expression when facing the victims (Abe et al., 2007; Baumgartner et al., 2009). This kind of emotional self-regulation has been found in fraudsters (Krokoszinski and Hosser, 2016) and contributes to their ability to remain undetected.

In addition to understanding how fraudsters avoid detection, we also need to know what methods fraudsters use to exploit others. For example, a successful fraudster could make adjustments according to others’ responses, especially emotional responses, to maintain the continuity and credibility of lies and achieve their goals (Wolfe and Hermanson, 2004; Krokoszinski et al., 2018). One study found that high emotional arousal (both negative and positive) may increase the susceptibility to fraud (Kircanski et al., 2018), which could be exploited as a fraud tactic by a good fraudster to persuade the victim to comply. This kind of tactic, affecting others’ emotions, referred to as the management of others’ emotions or emotional manipulation that has been studied in different groups for decades (Austin et al., 2007; Grieve and Mahar, 2010; Grieve and Panebianco, 2013; Abell et al., 2016). A self-report scale Managing the emotions of others scale (MEOS) was developed to measure people’s manipulation style (Austin and O’Donnell, 2013) to understand the fraudster’s behavioral traits. Nevertheless, this style of manipulation is rarely explored in real fraudsters. Thus, to protect people against fraud, it is necessary to deepen the understanding of how fraudsters regulated the victim’s emotions in the process of deception.

The present study aims to explore the emotional manipulation, psychopathy, and emotion recognition in imprisoned fraudsters that would help a better prediction of the social interaction between fraudsters and victims during a fraud event. As the function of empathy in social interaction, we also want to explore the difference in empathy between the imprisoned fraudster and the control group. We hypothesized that (1) in comparison to controls, imprisoned fraudsters would have a higher accuracy of emotion recognition and a better ability to manage others’ emotions, and (2) imprisoned fraudsters would have a higher level of empathy than the control group. Psychopathy is closely related to manipulative behavior (Babiak, 1995; Hare, 2003; Babiak et al., 2006; Gao and Raine, 2010; Schouten et al., 2012) and it was prevalence in prison (Ullrich et al., 2003; Assadi et al., 2006). (3)We would assess the psychopathy level of imprisoned fraudsters, and it was expected to be higher than the control group.

## Materials and Methods

### Participants

Previous research about fraudster’s ability to recognize others’ emotions (Krokoszinski et al., 2018) had an effect size (ES) of *Cohen’s d* = 1 that was a medium-level ES (Ferguson, 2016). We set an ES *Cohen’s d* = 1 with 80% power (*α* = 0.05, two-tailed) in G^∗^Power (Faul et al., 2007), which suggested we need 17 participants in each group (*N* = 34) in an independent sample *t*-test. According to the current study’s aim, each fraudster must have at least one face-to-face fraud conviction. After checking their official criminal records, 34 fraudsters met this criterion and agreed to participate in the experiment. Concerning detailed criminal history, most of these 28 fraudsters involved contract fraud between companies, such as one company paid down a deposit, and then the criminals diverted it for other purposes. The rest were scams between acquaintances, such as pretending to promise to buy goods for them. Five had previous convictions, and the others were first-time inmates. The demographics inclusion criteria were as follows: (1) age from 18 to 45 years old; (2) no neurological illness, head trauma, substance abuse, or dependence. The healthy control group (*n* = 31) was recruited from the community through online announcements. The two groups were matched in age, education level, and IQ (see Table 1) using the Chinese version of Standard Progressive Matrices (SPM; Zhang, 1989).

**TABLE 1:** Demographic information of two groups (*M* ± *SD*).

	Fraudster	Control	*t*	*p*
	*N* = 34	*N* = 31		
Age	36.82 (6.54)	35.06 (9.88)	0.84	0.41
Education level (years)	10.20 (3.38)	9.71 (1.34)	0.79	0.43
SPM raw score	38.76 (9.39)	41.65 (8.67)	1.36	0.18

All participants took part in the study voluntarily and provided written informed consent. The offenders received daily necessities, and the control group received 50¥ for their participation. The study was approved by the Prison Administration Bureau of the Ministry of Justice of China and the Institutional Research Ethics Committee of China University of Political Science and Law (No. 2018072201).

### Materials

#### Emotional Recognition Task

**FIGURE 1:**
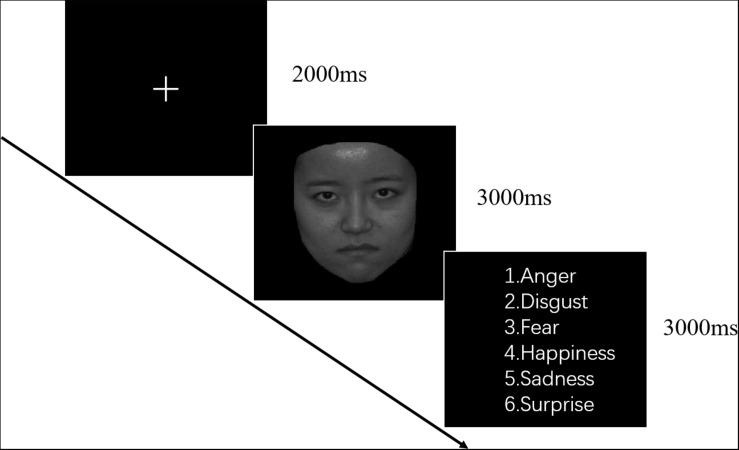
Example of stimuli used in the emotion recognition task.

Sixty pictures of Chinese faces that contained six basic emotions were selected from a standardized emotion system (Gong et al., 2011). Six emotions (anger, disgust, fear, happiness, sadness, and surprise) were depicted by ten actors (five males and five females). All stimulus materials were presented on a 27-inch computer via E-prime 2.0 (Psychology Software Tools, Inc.). Each picture appeared in the middle of the screen and was only presented once for each participant. The task is illustrated in Figure 1. Each picture was presented for 3s, followed by a prompt to identify the emotional category (3s). Participants responded through a keyboard by button press.

#### Managing the Emotions of Others Scale

Managing the Emotions of Others Scale is developed to measure the ability of emotional manipulation that could improve and worsen others’ emotions, which is a critical component of emotional intelligence (Austin and O’Donnell, 2013). Fifty-eight items combine into six factors (Enhance, Worsen, Conceal, Inauthentic, Poor skills, Divert) in MEOS. Among these factors, “prosocial” (Enhance/Divert) and “non-prosocial” (Worsen/Inauthentic) pair were the preferred ways of managing others’ emotions. The non-prosocial pair is regarded as the “dark side” of emotional intelligence (Austin et al., 2007; Kilduff et al., 2010; Petrides et al., 2011) because it could be used by instigators to induce negative emotions of others for specific behaviors they want. A Likert-style response was adopted in MEOS, ranging from 1 (completely disagree) to 5 (completely agree). We used the Chinses version of MEOS in the current study. The translation steps adopt the standard procedure suggested by Brislin (1986).

#### The Psychopathic Personality Inventory – Short Form

All participants completed the short form of the Psychopathic Personality Inventory (PPI-SF) to assess psychopathy. PPI-SF was a 56 items self-report scale and included eight factors: Blame Externalization, Social Potency, Machiavellian Egocentricity, Fearlessness, Impulsive Non-conformity, Carefree Non-planfulness, Coldheartedness, and Stress Immunity (Kastner et al., 2012). The Chinese version of PPI-SF was used in the current study (Gao, 2011).

#### Interpersonal Reactivity Index

The Interpersonal Reactivity Index assesses the dispositional empathic traits (IRI), which has four factors (Perspective Taking, Fantasy, Empathic Concern, Personal Distress) that consist of 28 items (Davis, 1983). IRI is a five-point scale ranging from 0 (completely disagree) to 4 (completely agree). The Chinese version of IRI (IRI-C) was applied in this study (Zhang et al., 2010).

#### Procedure

The author of this article conducted all steps of the experiment. The incarcerated offenders’ data were collected at a single quiet room (used for offenders’ daily education) in a prison in northwest China. When the experimenter carried out the experiment, a policeman was waiting outside the door in case there was a security problem. Offenders were told that their data would be kept anonymous and would be confidential. The control group participated in the experiment in a laboratory at the China University of Political Science and Law.

After the screening process and completing the informed consent, offenders completed the self-report scales (MEOS, PPI-SF, and IRI-C) and an emotion recognition task. The control group followed the same protocol. After completing the study, participants were remunerated.

#### Statistical Analysis

Data analysis was conducted using SPSS 23.0. Independent *t*-tests were used to compare the differences in the emotion recognition (ER) task and self-report scales between the incarcerated offenders and control groups. Finally, Pearson’s correlation was used to explore the association between ER, PPI-SF, MEOS, and IRI-C in each group.

## Results

### Emotion Recognition Accuracy

**FIGURE 2:**
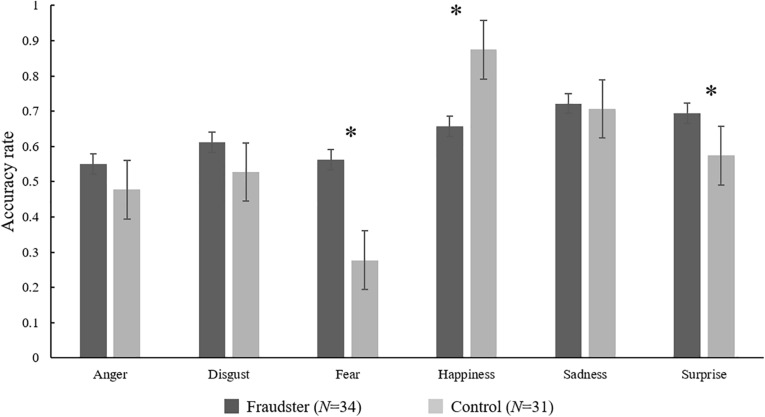
Mean accuracy rate of emotions for fraudsters and control groups. *p < 0.05.

The *t*-test revealed a significant difference in the accuracy of emotion (Figure 2). The results show that imprisoned fraudsters had higher accuracy in fear and surprise (*t* = 5.26, *p* < 0.001, *d* = 1.31; *t* = 2.38, *p* = 0.02, *d* = 0.59) than the control group but lower in happiness (*t* = 3.75, *p* < 0.001, *d* = 0.93).

### Self-Report Scales Analysis

A *t*-test was used to compare two self-report scales between the two groups (see Table 2). Results showed that the control group gained a higher score than the imprisoned fraudsters on Inauthentic, Worsen, non-prosocial, and MEOS total score (*all ps* < 0.01). The imprisoned fraudsters were scored lower than the control group in most PPI-SF factors, except Carefree Non-planfulness and Coldheartedness (*ps* < 0.05). For empathy, the incarcerated fraudsters scored higher than the control group in PT (*p* < 0.05).

**TABLE 2:** Results of comparing two groups on MEOS, PPI-SF, and IRI-C.

	Fraudster	Control	*t*	*p*	Cohen’s *d*
	*N* = 34	*N* = 31			
**MEOS**					
*Enhance*	52.73 (9.63)	53.32 (9.37)	0.25	0.80	0.06
*Worsen*	29.55 (9.72)	34.58 (5.93)	2.54	**0.01**	0.62
*Conceal*	22.11 (4.48)	22.68 (3.44)	0.56	0.58	0.14
*Inauthentic*	25.85 (8.44)	32.74 (6.66)	3.63	**< 0.01**	0.91
*Poor skills*	26.14 (4.30)	26.87 (3.78)	0.72	0.47	0.18
*Divert*	24.82 (4.35)	26.07 (4.61)	1.12	0.27	0.28
*Non_Prosocial*	55.41 (17.55)	67.32 (11.39)	3.21	**< 0.01**	0.81
*Prosocial*	77.55 (12.75)	79.39 (12.96)	0.57	0.57	0.14
*MEOS total*	162.14 (36.64)	196.26 (19.30)	4.75	**< 0.001**	1.17
**PPI-SF**					
*Blame*	12.91 (3.18)	15.80 (3.19)	3.65	**< 0.01**	0.91
*Social*	17.52 (3.44)	16.29 (2.26)	0.96	0.34	0.42
*Machiavellian*	15.20 (3.75)	16.93 (3.37)	1.95	0.06	0.49
*Fearlessness*	15.35 (3.46)	19.41 (3.80)	4.51	**< 0.001**	0.12
*Impulsive*	14.11 (3.17)	16.22 (3.01)	2.74	**< 0.01**	0.68
*Stress*	17.64 (3.02)	17.09 (2.35)	0.81	0.42	0.20
*Carefree*	18.76 (4.94)	14.51 (2.70)	4.24	**< 0.001**	1.07
*Coldheartedness*	21.85 (7.07)	16.67 (2.21)	3.90	**< 0.001**	0.99
*Factor 1*	50.52 (7.06)	53.35 (3.77)	2.04	**0.05**	0.50
*Factor 2*	61.00 (11.84)	63.48 (7.68)	0.99	0.33	0.25
*PPI-SF total score*	133.32 (18.82)	133.64 (9.95)	0.09	0.93	0.02
**IRI-C**					
*EC*	16.56 (3.24)	16.94 (3.27)	0.47	0.64	0.12
*PT*	17.47 (3.59)	15.81 (2.94)	2.03	**0.05**	0.51
*FS*	12.79 (5.57)	14.29 (3.80)	1.25	0.22	0.31
*PD*	14.97 (4.48)	15.94 (3.29)	0.98	0.33	0.25
*IRI-C total*	61.85 (12.72)	62.97 (7.73)	348.5	0.44	0.68

*PT, perspective-taking scale; FS, fantasy scale; EC, empathic concern scale; PD, personal distress scale.*

### Correlation Analysis

#### Relationship Between ER, MEOS, and IRI-C

To further examine the relationship between MEOS and PPI-SF, ER, and IRI-C, Pearson correlation analyses were conducted separately within the groups (Tables 3, 4). In both groups, most PPI-SF factors, including Factor 1, Factor 2, and the total score, were only positively correlated with non-prosocial factors of MEOS. However, there was no consistent result in the correlation between ER/IRI-C and MEOS in the two groups.

**TABLE 3:** Correlation results between MEOS and PPI-SF, ER, and IRI-C in fraudsters (*N* = 34).

	Enhance	Worsen	Conceal	Inauthentic	Poor skills	Divert	Non-prosocial	Prosocial	MEOS-total
**PPI-SF**									
*Blame*	–0.15	0.62**	0.37*	0.55**	0.43*	–0.21	0.61**	–0.19	0.42*
*Social*	0.24	0.09	–0.04	0.04	0.13	0.20	0.07	0.25	–0.04
*Machiavellian*	0.22	0.82**	0.48**	0.73**	0.53**	0.02	0.81**	0.17	0.65**
*Fearlessness*	0.15	0.65**	0.36*	0.63**	0.37*	0.20	0.66**	0.18	0.36*
*Impulsive*	0.22	0.57**	0.47**	0.49**	0.56**	0.03	0.56**	0.17	0.46**
*Stress*	0.29	0.02	0.41*	0.07	0.04	0.28	0.05	0.32	0.06
*Carefree*	0.17	0.27	0.14	0.10	0.16	–0.12	0.20	0.09	0.61**
*Cold*	–0.11	0.07	–0.04	0.03	0.15	0.09	0.06	–0.05	−0.53**
*Factor 1*	0.32	0.37*	0.33	0.36*	0.26	0.32	0.38*	0.35*	0.18
*Factor 2*	0.16	0.70**	0.43*	0.55**	0.50**	–0.09	0.65**	0.09	0.69**
*PPI-SF total*	0.18	0.60**	0.38*	0.50**	0.47**	0.10	0.57**	0.17	0.31
**ER**									
*Anger*	–0.04	–0.07	0.12	–0.14	–0.04	0.10	–0.10	0.01	0.01
*Disgust*	–0.02	–0.10	0.14	–0.26	–0.09	–0.12	–0.18	–0.06	0.05
*Fear*	0.01	–0.30	0.11	−0.43*	–0.14	–0.03	−0.37*	–0.01	–0.15
*Happiness*	0.34*	–0.14	0.15	–0.22	–0.02	0.02	–0.18	0.27	0.22
*Sadness*	0.01	0.01	0.08	0.11	–0.31	0.02	0.06	0.02	0.18
*Surprise*	0.16	–0.16	0.02	–0.27	–0.16	0.16	–0.22	0.17	0.03
**IRI-C**									
EC	–0.23	0.10	0.08	0.13	–0.17	–0.09	0.12	–0.21	0.10
PT	0.41*	0.08	0.21	0.05	–0.18	0.12	0.07	0.35*	0.38*
FS	0.03	0.26	0.09	0.26	0.17	–0.14	0.27	–0.02	0.31
PD	−0.37*	0.29	0.05	0.34	–0.05	–0.17	0.32	−0.34*	0.04
IRI-C total	–0.06	0.27	0.14	0.28	–0.04	–0.11	0.28	–0.08	0.29

PT, perspective-taking scale; FS, fantasy scale; EC, empathic concern scale; PD, personal distress scale. *p < 0.05. **p < 0.01.

**TABLE 4:** Correlation results between MEOS and PPI-SF, ER, and IRI-C in the control group (*N* = 31).

	Enhance	Worsen	Conceal	Inauthentic	Poor skills	Divert	Non-prosocial	Prosocial	MEOS-total
**PPI-SF**									
*Blame*	0.10	0.30	0.32	0.35	0.28	0.25	0.36*	0.16	0.44*
*Social*	−0.48**	0.20	–0.11	0.25	−0.62**	–0.11	0.25	0.39*	–0.25
*Machiavellian*	0.17	0.32	0.47**	0.55**	–0.12	0.23	0.49**	0.21	0.49**
*Fearlessness*	0.19	0.39*	0.15	0.55**	0.19	0.28	0.53**	0.24	0.53**
*Impulsive*	0.07	0.26	0.38*	0.08	−0.37*	0.33	0.18	0.17	0.21
*Stress*	0.05	–0.16	0.07	–0.32	–0.16	–0.01	–0.27	0.03	–0.17
*Carefree*	−0.52**	–0.21	−0.62**	–0.12	–0.01	−0.39*	–0.18	−0.51**	−0.57**
*Cold*	0.69**	0.12	0.47**	0.04	0.04	0.48**	0.09	0.67**	0.59**
*Factor 1*	–0.07	0.41*	0.10	0.51**	–0.29	0.22	0.51**	0.03	0.28
*Factor 2*	–0.04	0.29	0.27	0.37*	–0.09	0.20	0.37*	0.04	0.28
*PPI-SF total*	0.10	0.40*	0.35	0.49**	–0.17	0.34	0.49**	0.19	0.45*
**ER**									
*Anger*	0.10	0.02	0.18	–0.09	0.06	–0.11	–0.04	0.03	0.04
*Disgust*	0.39*	0.14	0.24	–0.17	0.05	0.23	–0.03	0.36*	0.28
*Fear*	0.15	0.14	0.19	0.12	0.13	–0.01	0.14	0.11	0.21
*Happiness*	0.11	–0.09	0.12	–0.11	–0.01	–0.31	–0.11	–0.03	–0.06
*Sadness*	0.23	–0.10	0.22	–0.12	0.08	–0.15	–0.12	0.12	0.06
*Surprise*	0.12	–0.18	0.13	–0.18	–0.15	–0.03	–0.20	0.08	–0.07
**IRI-C**									
EC	0.34	–0.30	0.09	–0.21	–0.18	0.06	–0.28	0.27	–0.01
PT	0.39*	–0.23	0.23	−0.43*	–0.15	0.19	−0.37*	0.35	0.03
FS	0.01	0.10	–0.02	0.20	0.05	–0.10	0.17	–0.03	0.09
PD	–0.06	0.13	0.03	0.16	0.13	–0.19	0.16	–0.11	0.05
IRI-C total	0.27	–0.11	0.13	–0.09	–0.05	–0.03	–0.11	0.19	0.07

PT, perspective-taking scale; FS, fantasy scale; EC, empathic concern scale; PD, personal distress scale. *p < 0.05. **p < 0.01.

## Discussion

This study was designed to examine emotional manipulation and its associations with psychopathy, emotional recognition, and empathy in incarcerated fraudsters. As predicted, the fraudsters performed better for negative emotion recognition. Fraudsters recognized fear and surprise with significantly higher accuracy than the control group while identifying happiness with lower accuracy than the control group. Regarding emotional manipulation, imprisoned fraudsters had lower scores in the non-prosocial pair of MEOS, including Worsen and Inauthentic subscales and the MEOS total score. However, they did not differ from the control group in prosocial factors. For psychopathy, imprisoned fraudsters had lower scores in PPI-SF factors, including Factor 1 and Factor 2. Results for empathy indicated that the fraudsters scored higher than the control group in Perspective Taking of IRI-C. Further correlation analysis revealed that psychopathy was mainly associated with non-prosocial factors. The negative emotions, such as disgust and fear, had different correlations with emotional manipulation in both groups. The correlation between empathy and emotional manipulation was different in the two groups.

Concerning emotional manipulation, the fraudster group showed low non-prosocial tendencies, the opposite of what we expected. Such lower scores on non-prosocial scales suggested that fraudsters tend to avoid mood-worsening behaviors, such as criticism/negative comments or displaying anger to manipulate others. The explanation for this phenomenon should consider that most imprisoned fraudsters in our study were charged with contract fraud. They may prefer to avoid contentious or negative interactions that might interfere with or draw attention to their scheme. In contrast, the prosocial manipulation would enhance the possibility of being trusted, which also precisely corresponded to the similarity of prosocial factors scores in MEOS between the two groups. Our study did not support the previous findings that fraudsters showed prosocial tendencies such as gregariousness, outgoingness, and agreeableness (Nee et al., 2019), but at least they are less prone to antisocial emotional manipulation. However, this antisocial avoidance in fraudsters could reflect a self-report bias because they may not want to display socially undesirable non-prosocial behavior when surveyed. Actually, fraudsters may still use non-prosocial manipulation strategies to adapt to their situation. If the victims were their subordinates, non-prosocial manipulation might be adopted. Previous research found that fraudsters leverage trust or authority to cheat or commit fraud (Shover and Hochstetler, 2005).

The fraudsters were not associated with greater psychopathy than control. PPI-SF results showed that the fraudsters had a lower level of psychopathy than the control group, which is different from previous studies (Blickle et al., 2006; Boddy, 2006; Ray, 2007; Bucy et al., 2008; Price and Norris, 2009). The fraudsters showed conscientiousness, such as self-discipline, and acted dutifully in their work (Blickle et al., 2006). Some white-collar criminals with psychopathy who committed fraud could even control their impulses by monitoring their behavior (Burkely, 2010) and thinking through decisions (Simpson and Piquero, 2002). These results suggested that not all fraudsters were associated with psychopathy, though it was regarded as a risk factor in many pieces of research. Fraud crime requires a well-planned script and careful implementation, representing a more complicated activity than the most violent or street crime. These make them hard to be detected; most of them committed multiple crimes but were only caught one time (e.g., only five offenders in the current study had previous conviction). Therefore, psychopathy cannot explain the full extent of fraud.

Fraudsters showed better recognition for negative emotions (fear and disgust) and lower accuracy in detecting happiness than control. This result is in line with previous studies in which fraudsters showed high sensitivity when detecting others’ emotions (Möller, 2009; Krokoszinski et al., 2018). Other research has identified a subgroup of fraudsters that may adopt violent behavior to prevent themselves from being detected and exposed (Perri and Lichtenwald, 2007; Brody and Kiehl, 2010), making them more accustomed to expressions of fear and surprise than others (Kirsh and Mounts, 2007; Diaz et al., 2016). For the emotion of happiness, however, fraudsters had lower accuracy than the control group. The first reason was that the fraudsters achieved their goal, usually through bullying or violence, which led to their rarely seeing others’ happy faces. This reduction of happiness accuracy also appeared in people who play violent games (Kirsh and Mounts, 2007). The second reason was the distrust of others in daily life. It was hard to imagine a liar trusting others easily, especially after living in a complicated environment like a prison for years.

For empathy, the fraudsters hold a better ability to understand others’ points of view. Results suggested that perspective-taking may be a critical skill when conducting a successful fraud. Criminals who can infer their victims’ emotions might be better able to react appropriately to their target and maximize their interests. This may relate to why the fraudsters in this study have a history of successful contract fraud. Nevertheless, with the limited sample and inadequate empathy measures, we need more studies and approaches to characterize empathy in fraudsters and obtain credible conclusions.

Correlation analysis was conducted to explore further the relationships between emotional manipulation and emotion recognition, psychopathy, and empathy. However, only psychopathy traits were found to have more positive correlations with non-prosocial manipulation across the groups, which was partly consistent with previous studies (Grieve and Mahar, 2010; Austin and O’Donnell, 2013). This positive correlation suggests that people with psychopathy usually have an antisocial lifestyle (Hare, 2003), and non-prosocial manipulation is one of its manifestations. Nevertheless, what remains to be tested is whether psychopathy is only associated with non-prosocial manipulation and why the fraudsters show low non-prosocial manipulation. Future studies could try to explore the emotional manipulation in psychopathic individuals.

The present study had some limitations. Replication with a larger sample size would help bolster confidence in the observed associations in the future study. Secondly, the ecological validity of the task paradigm should be improved. More effective measurements for emotional manipulation should be developed. We only used the self-report scale to measure emotional manipulation, which would easily lead to bias responses when filling the scale. Thirdly, other types of fraudsters should also be explored. The fraudsters in this study were mainly face-to-face frauds. The characteristics of telecom fraudsters are still unknown, which is the most popular form of fraud. Finally, more personality traits, such as Machiavellianism and narcissism, should be considered because they were closely associated with manipulation (McHoskey, 1995; Rauthmann and Will, 2011).

In summary, this study provided valuable contributions to the exploration of emotional manipulation in incarcerated fraudsters, and the unique characteristics of fraudsters were found. Specifically, the present study indicates that the fraudster was not associated with psychopathy; they have better emotion recognition ability and avoid using non-prosocial methods of manipulation. Although not exhaustive, these results offer insight into factors contributing to fraudster behavior.

## Data Availability Statement

The raw data supporting the conclusions of this article will be made available by the authors, without undue reservation.

## Ethics Statement

The studies involving human participants were reviewed and approved by the Institutional Research Ethics Committee of China University of Political Science and Law. The patients/participants provided their written informed consent to participate in this study. Written informed consent was obtained from the individual(s) for the publication of any potentially identifiable images or data included in this article.

## Author Contributions

QLW wrote the manuscript and managed the study. QLW, ZLL, and SLB collected and analyzed the data. ZZ, BY, and CL edited the manuscript and conceived of the research design, methods, and analyses. ZBL, ED, and AV helped interpret data and improve writing. All authors contributed to the article and approved the submitted version.

## Conflict of Interest

The authors declare that the research was conducted in the absence of any commercial or financial relationships that could be construed as a potential conflict of interest.
